# A narrative review of papilla preservation techniques in clinical dentistry

**DOI:** 10.1097/MD.0000000000041033

**Published:** 2025-01-17

**Authors:** Yinghua Fu, Zhixin Zhang, Xiaoping Tang, Jiangling Su

**Affiliations:** a Department of Stomatology, Quanzhou First Hospital Affiliated to Fujian Medical University, Quanzhou, Fujian, China.

**Keywords:** aesthetics, clinical efficacy, incision design, papilla preservation, periodontal healing

## Abstract

The interdental papilla is closely associated with oral health and dental aesthetics. Interproximal papilla is an essential component of pink aesthetics as well as an indispensable prerequisite for the health of oral tissues. The loss of papillary height not only considerably affects final esthetic results, but also brings a series of periodontal complications. The deficiency of papilla can be induced by iatrogenic factors, particularly the flap technique, which is usually employed in oral surgeries. Therefore, preservation of the interdental papilla must be a consideration of flap designs. To avoid papilla defects secondary to conventional flaps, papilla-preserving flap designs have been widely studied in clinical practice. With numerous papilla preservation techniques (PPTs) increasingly being employed, not only is the postoperative pink aesthetic better maintained, but the predictability of surgical outcomes has been significantly enhanced. However, there is a lack of adequate literature that provides a comprehensive overview of PPTs in the field of dentistry. This review summarizes the latest developments in papilla preservation flap designs in the field of oral surgery, with a particular emphasis on their indications, limitations, incision characteristics, and clinical benefits. This review may provide optimal protocols for the personalized treatment.

## 1. Introduction

In a healthy periodontium, the gingival papilla fills the interdental space, the space beneath the contact area of 2 adjacent teeth. Gingival papilla recession refers to the apical shift of the gingival free margin in the interproximal region. This recession often results in a dark triangle apical to the contact area. When the dark triangle is present, esthetic concerns and phonetic difficulty may occur. Other complications include root sensitivity, plaque accumulation, food impaction, root caries and periodontal inflammation.^[1]^ Both oral function and dental aesthetics are main treatment objectives in modern dentistry, therefore, the prevention of papillary recession has become an urgent clinical focus.

The loss of papilla is associated with non-iatrogenic factors, such as periodontal phenotype, age-relative recession, gingival biotype, contact point height and shape of the crown.^[2–4]^ Iatrogenic factors, like the surgical flap which is usually required in clinical practice, also contribute to this condition. Due to the papilla’s unique anatomical and physiological characteristics, containing narrow interproximal space, limited blood supply and individual differences, it cannot spontaneously be restored once retracted.^[5]^ With the advent of microsurgical instruments and 3-dimensional printing techniques, dental operations have become more precise and less invasive, allowing better retention of the interdental papilla. In addition to the maintenance of papillary height by papillary preservation techniques, mounting evidence supports of their capacity to enhance the predictability of surgical outcomes. Nevertheless, previous reviews on papilla preservation flap designs are limited, failing to provide a systematic overview of their applications in different clinical scenarios. This review will focus on the effect of different surgical techniques on papilla preservation and the evolution of surgical efficacy from the perspective of flap design, including those used in endodontic, periodontal, implant, alveolar surgery, and accelerated periodontal osteogenesis orthodontics.

### 1.1. Papilla preservation flap designs in endodontic surgery

A good soft tissue contour is critical to the aesthetic success of endodontic surgery. The flap design plays a role in gingival recession, attachment loss, increased probing depth, and exposure of the edge of the restoration, all of which can damage the “pink and white aesthetic.” Traditional flap designs, like envelope flap, triangular flap, and rectangular flap, include an intrasulcular incision (ISI) surrounding at least 2 teeth. Although these techniques have advantages in surgical access and scar-free healing, there remain limitations in gingival margin recession and crestal bone resorption.^[6–8]^ The effects of ISI on periodontal parameters remain controversial. Some studies have confirmed that ISI can reduce the papillary height to varying degrees. As shown in Table 1, several incision designs have been recommended to preserve the papilla, including the papilla base incision (PBI), the submarginal incision (SMI), and the semilunar incision methods.^[9]^ Velvart et al^[10]^ conducted a study to evaluate papilla recession after apical surgery with ISI and PBI, independently. The results demonstrated that ISI led to a significant decrease (0.98 ± 0.75 mm) in the papillary height at 1 year of follow-up, whereas papilla recession was <0.1 mm in the PBI group. A different cohort study revealed that none of the 3 flap designs resulted in significant loss of postoperative papilla height, instead reporting stagnant baseline crest bone height via cone-beam computed tomography (CBCT) at 18 month follow-up.^[11]^ These contrasting findings may be attributed to differences in exclusion criteria, sample size, or case selection criteria.

**Table 1:** Papilla preservation flap designs in endodontic surgery.

Incision types	Incision design			Incision characteristics	Indications	Limitations
	Gingival papilla base	Attached gingiva	Releasing incision			
Papilla base incision	+	−	+	The horizontal incision consists of intrasulcus and papillary base incision. The papillary base incision contains a first incision perpendicular to the papilla and a second incision directing to the alveolar crest.	Apical lesion extending to the coronal portion of the root	① High technical sensitivity ② prolonged surgical time
Submarginal incision	−	+	+	The scalloped horizontal incision is located between the base of gingival sulcus and mucogingival junction, extending 1 tooth medially and distally to the involved tooth.	① Attached gingiva beyong 2 mm ② apical lesion not involving cervical portion of the root	① Preoperative measurement of gingival sulcus depth is required. ② suture is time-consuming and poor suture tends to leave scars.
Semilunar incision	−	−	−	The incision is at least 5 mm away from the bone defect area, convex in marginal direction. Extends 1 adjacent tooth around the target tooth, and avoid the frenulum.	Lesion confined to the apical zone.	① Excessive bleeding in the surgical area and poor visualization of the surgical field ② greater postoperative swelling and pain ③ tendency to poor healing and scar formation.

Notes: Releasing incision: Starts from between apical third and middle third of the papilla, the initial direction is perpendicular to the gingival margin, and subsequently turns parallel to the long axis of the tooth towards the vestibule; “ + ” represents corresponding incision, “ − ” represents no corresponding incision.

However, poor suturing of the gingival flaps is susceptible to scar formation, which may be an aesthetic risk for PBI and SMI. Endodontic microsurgery, which has improved success rates of apical surgery to 90%,^[12]^ utilizes tension-free alignment sutures on the wound margins and promotes migration and crawling of epithelial cells during the healing process, thereby reducing the incidence of scar formation. Velvart et al^[13]^ performed apical microsurgery with PBI, observing scarless healing across most incision lines 1 month post-operatively. In addition to affecting periodontal healing, the flap design was associated with the short-term inflammatory response and quality of life of patients. A randomized clinical trial demonstrated that the PBI group exhibited milder pain, expedited recovery, and higher quality of life scores compared with the ISI group 1 week after apical microsurgery.^[14]^ Dimova et al^[15]^ reported that the patients using a semilunar incision technique experienced less postoperative pain than those undergoing ISI, though they presented more difficulty in mouth opening, mastication, and speaking. In contrast, Ahmed et al^[16]^ observed that although the pain in traditional flap technique was higher than that of SMI, it was not statistically significant. Available studies are limited, and sample size with them is relatively small. It seems that ISI could unfavorably decrease the patient’s quality of life more than other incision types.^[17]^ While PBI has improved the postoperative experience, the impact of PBI on aesthetics is marginal as the free margin retraction on the midbuccal is not visible.^[6]^ Therefore, if the papilla height is conserved, PBI may be the best choice. Kirkevant et al^[18]^ endorsed that PBI should be adopted by clinicians with sufficient surgical experience, due to its higher technique sensitivity.

Modern endodontics has enabled more minimally invasive incision techniques. For instance, direct intra-oral identification of the root apex is extremely difficult with free hand surgery, inevitably leading to gingival flap elevation and alveolar bone fenestration. The use of preoperative 3D information as a guide in endodontic microsurgery allows patient-specific treatment, significantly reducing surgical time and the volume of bone preparation, improving postoperative healing. Tu et al^[19]^ carried out an apicoectomy with a minimally invasive straight incision in the vestibular mucosa (the incision parallel to the tooth axis, with a length <1 mm) under the application of a guide, demonstrating satisfactory recovery of the periapical lesion and mucosal wounds after surgery. A similar study by Kim et al^[20]^ conducted an apical microsurgery on anterior teeth of root canal calcification with a 3D guide template. The mucosa was only incised at the access site, and no periodontal soft tissue complications occurred at 1 month follow-up. Due to the constrained visualization of the straight incision, it is necessary to strictly restrain its indications. Only when the lesion is limited and can it be accurately located with the surgical guide, and a minimally invasive straight incision utilized. Complete exposure and removal of lesions should be given priority over excessive pursuit of minimally invasive techniques.

### 1.2. Papilla preservation flap designs in periodontal surgery

**Figure 1.**
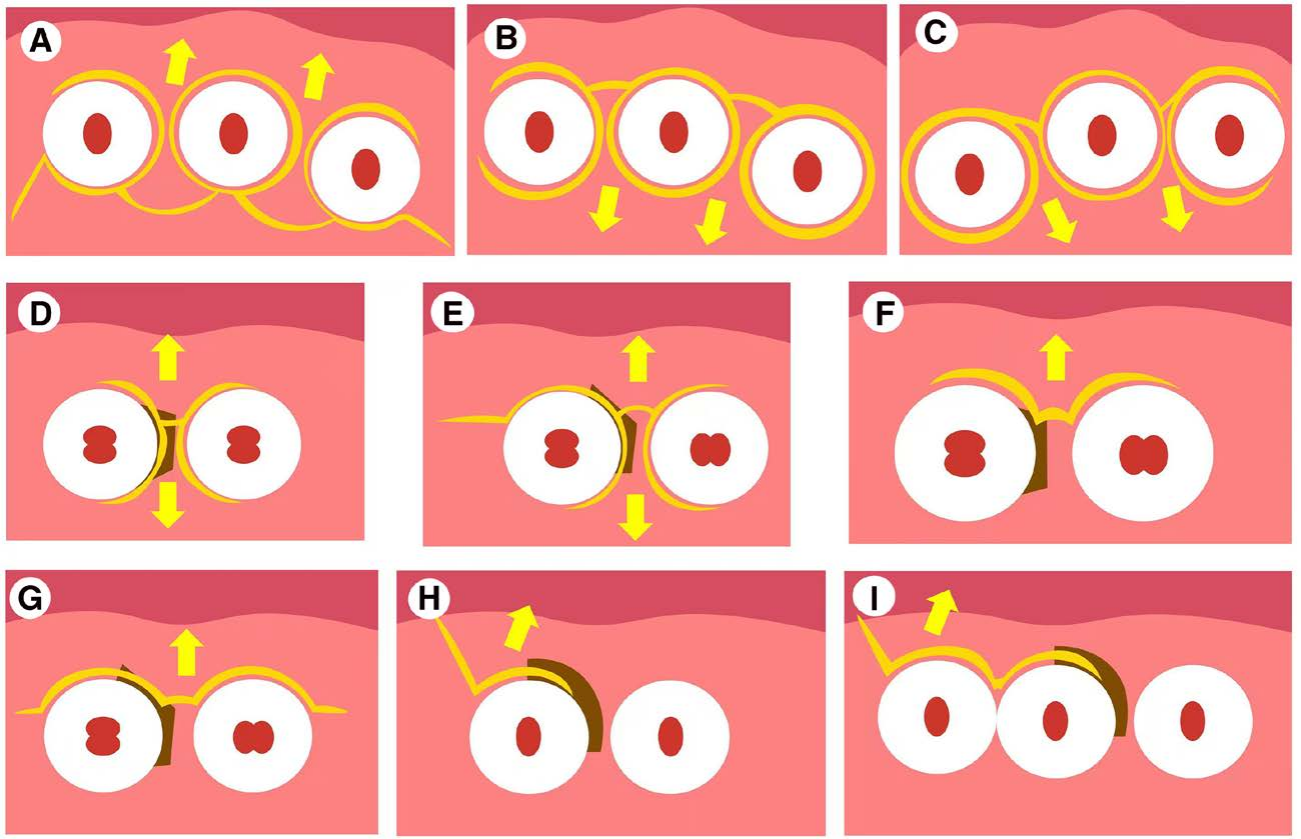
Illusion of flap designs in periodontal surgery (occlusal view of cervical cross section). (A) papilla preservation technique; (B) modified papilla preservation technique; (C) simplified papilla preservation technique; (D and E) minimally invasive surgical technique; (F) modified minimally invasive surgical technique; (G) single flap approach; (H and I) entire papilla preservation technique. Note: The yellow arrow indicates the direction of flap reflection, and the brown area indicates osseous defects.

Traditional periodontal surgery is prone to postoperative gingival recession, a concave shape at the coronal point of the papilla leading to plaque retention and persistent inflammation of soft tissue.^[21,22]^ Moreover, in the interdental papilla, blood supply is limited and tension-free closure in the early stage is hard, making it difficult to regenerate periodontal tissue in the interproximal space. Previous studies have shown that wound dehiscence and exposure of the barrier membrane usually occur following guided tissue regeneration surgery (GTRS), which is detrimental for the survival of bone graft materials, especially in the interdental area.^[23,24]^ To attain a more predictable result of bone augmentation in the interdental area, the papilla preservation technique (PPT) was proposed by Takei et al^[25]^ in 1985 (Fig. 1A). A semilunar incision is placed 5 mm from the palatal/lingual papilla, subsequently the intact interdental papilla is attached to the buccal/labial flap and elevated to the buccal/labial side through the interdental space. However, when more bone grafting space is needed, the palatal flap may not be able to coronally cover the bone grafting area with sufficient tension. Therefore, researchers have proposed the modified papilla preservation technique (MPPT) (Fig. 1B) and simplified papilla preservation technique (SPPT) (Fig. 1C), based on PPT.^[26,27]^ The semilunar incisions on the palatal papilla have been substituted with horizontal incisions on the labial papillary base and oblique incisions on the papilla, respectively. In addition, the SPPT expands the indications to posterior tooth area with narrow interproximal distance of <2 mm.

As more regeneration materials (such as enamel matrix derivatives) emerge, the barrier membrane is no longer a necessity in GTRS. Furthermore, advances in microscopy have created a clearer visual field of operation. In 2007, Cortellini and Tonitti^[28]^ proposed the minimally invasive surgical technique (MIST) (Fig. 1D and E), which strictly limits the flap range to the adjacent tooth line angle or continuously expands the surgical area to 1 more tooth position, in accordance with the osseous defect area. Both the buccal and palatal sides of the flap are reflected, and the reflection area only exposes the surrounding bone crest 1 to 2 mm away from the bony defect. SPPT (interdental distance ≤ 2 mm) and MPPT (interdental distance > 2 mm) are utilized in the interproximal papilla to retain the papilla height. This technique is often executed in conjunction with enamel matrix derivatives, though not suitable for covering barrier membranes. In 2009, Cortellini and Tonetti^[29]^ further improved MIST (Fig. 1F) by placing a sulcular incision on the buccal side of the papilla in the bone defect, elevating the labial/buccal flap while retaining the interproximal papilla and lingual/palatal flap. However, this procedure was restricted to cases where debridement could be accomplished through the labial/buccal approach. For intrabony defects that extend to the buccal or lingual side of involved tooth, barrier membranes may be more favorable for periodontal regeneration. In 2009, Trombelli et al^[30]^ introduced a single flap approach (SFA) (Fig. 1G), which reflects either the buccal or lingual flap while expanding the reflection range to accommodate barrier membrane. When thorough debridement is guaranteed, SFA may be superior to bilateral flap techniques.^[31,32]^

Although previous techniques have greatly reduced the damage to the papilla, the blood supply cannot be preserved completely. For isolated and deep intrabony defects, improper flap approaches may substantially reduce the treatment effectiveness. In 2017, Aslan et al^[33,34]^ proposed the entire papilla preservation technique (EPPT) (Fig. 1H and I). The main step of the surgery involves a releasing incision at the buccal line angle of the affected tooth away from the bony defect area, and an intrasulcus incision towards the defect area, permitting access to the bone defect area through a tunnel approach. Due to the reliance of the releasing incision on the supporting bone, space maintenance required for clot stabilization is further improved. EPPT could effectively avoid exposure of bone regeneration materials in the initial stage of healing.^[35]^ Similar to SFA, this procedure only elevates the gingival flap of 1 aspect and is not suitable for large intrabony defects on the lingual/palatal side.

The stable structure of 2-wall and 3-wall bony pockets is conducive to accommodating grafts, and studies have shown predictable periodontal regeneration in such bony defects. However, the prognosis of bone regeneration in teeth with single wall defect and mobility grade II or III is not yet clear, and there is high risk of soft tissue collapse and gingival margin shrinkage. In an effort to address the unfavorable soft tissue shrinkage, Rodríguez and Cafffesse^[36]^ described a non-incised papillae surgical approach (NIPSA) in 2018. The range of bony defect is first defined through CT pre-operatively. Only a horizontal or oblique incision is placed on the buccal mucosa as apically as possible from the periodontal defect, creating an apical approach to enter the defect. However, NIPSA has relatively poor surgical field visibility and cannot be used for intrabony defects with intact buccal bone. In addition, the debridement of the lingual aspect must be implemented blindly, thereby increasing the risk of inflammatory granulation omission. Moreover, the apical incision may disrupt the blood supply from the apex, making NIPSA less effective than EPPT in preserving the blood supply to papilla.^[37]^

At present, RCTs on periodontal surgery have focused on the adjunctive efficacy of regenerative biomaterials combined with papilla preservation techniques, though biomaterials do not seem to result in additional clinical benefits as expected. When an ideal healing environment is available, the periodontal tissue attains self-healing potential and clinical improvement of surgical technique is independent of the use of regeneration materials.^[38–40]^

### 1.3. Papilla preservation flap designs in implant surgery

The reflection region of the flap attached to incision methods in implant surgery is critical for peri-implant papilla preservation. The flapless implant procedure is often preferred to preserve the papilla to the greatest extent.^[41]^ However, direct visualization of the bone width is not allowed, resulting in implant deviation. Moreover, bone regeneration is not permitted simultaneously.

Given the influence of flap designs on soft tissue morphology, a number of principles must be considered when employing incision techniques in implant surgery: preserve the blood supply of soft tissue; avoid elevation of papilla; facilitate the soft- or hard-tissue augmentation; and enable tension-free suturing. A papilla-sparing incision in implant surgery was described by Greenstein and Tarnow.^[42]^ This incision technique is characterized by a horizontal incision along the midcrestal or palatal aspect of the ridge, which is terminated 1 mm from the adjacent teeth without papilla involvement, and bilateral vertical releasing incisions on the buccal. If needed to transpose the keratinized gingiva to the buccal, the horizontal incision can be adjusted palatally. When bone grafting is required, the vertical releasing incisions can be appropriately extended. This surgical procedure is applicable for maintaining soft tissue height in thin gingival biotypes. A prospective study reported that papillae-sparing incisions resulted in significantly less interproximal bone loss compared to flap elevation with papillae detachment (0.29 mm ± 0.38 vs 1.12 mm ± 1.14).^[43]^ Regrettably, the study did not measure the loss of papillary height around the implant. Although papilla-sparing incisions have not been suggested to form visible scars,^[44]^ the placement of vertical releasing incisions may result in damage of vascular anastomoses and frequent observation of sequels like gingival recession and scars in the esthetic zone.^[45,46]^

**Figure 2.**
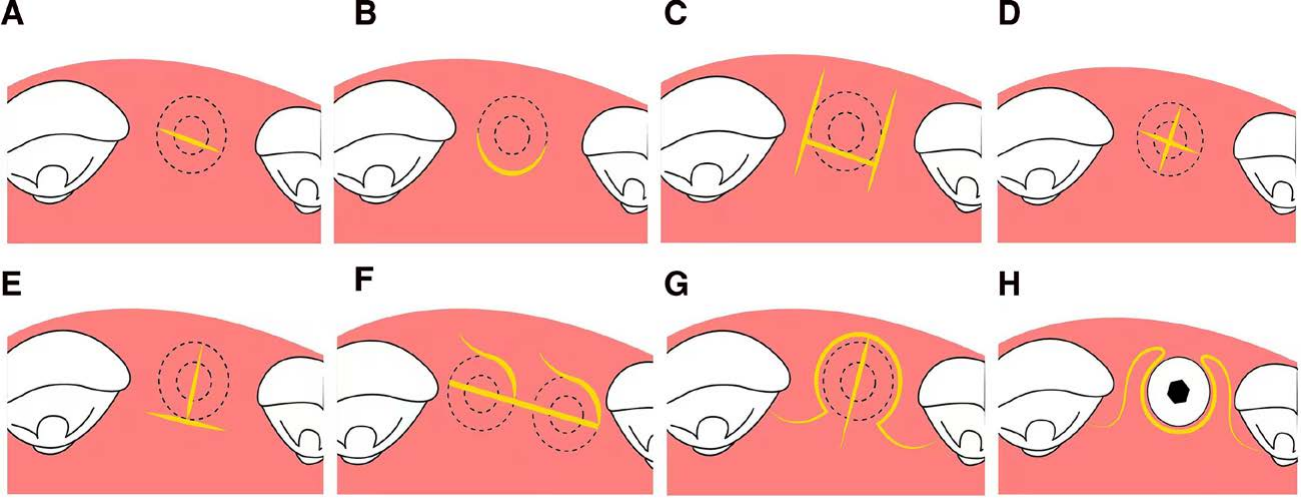
Illustration of papilla preservation incisions in 2-stage implant (occlusal view). (A) straight incision; (B) U-shaped incision; (C) H-shaped incision; (D) cross-shaped incision; (E) modified cross-shaped incision; (F) S-shaped incision; (G and H) split-finger incision.

Submerged implant protocol requires a 2-stage approach to expose the top of the implant after osseointegration, allowing the soft tissue to form a natural gingival cuff around the abutment. The 2-stage operation should obtain the necessary approach and aesthetics with minimal trauma and exposure. As shown in Table 2, the 2-stage surgical incisions with papilla preservation are divided into straight incision, U-shaped, H-shaped, cross-shaped, modified cross-shaped, S-shaped, C-shaped, and split-finger incisions (Fig. 2).^[47]^ A retrospective study by Luo et al^[48]^ verified that for 2-stage surgery, papilla-sparing incisions have a distinct advantage in the prevention of papilla atrophy compared with intrasulcular incisions.

**Table 2:** Papilla preservation incisions in 2-stage implant.

Incision designs	Incision characteristics	Indications
straight incision	The horizontal incision is along the midcrestal or palatal side of the ridge, with a short length.	① The diameter of healing abutment is short; ② the soft tissue in buccal aspect is adequate.
U-shaped incision	An arc-shape incision is set on the palatal side of the ridge, convex in palatal direction.	① The diameter of healing abutment is short; ② the soft tissue in buccal aspect is adequate.
H-shaped incision	The horizontal incision is along the palatal side of the ridge, and bilateral releasing incisions are 2 mm from the interproximal papillae.	① Implant in esthetic regions; ② implant location can be identified.
Cross-shaped incision	The horizontal incision is along the midcrestal or palatal side of the ridge, and terminated 2 mm from the adjacent papillae.	① Implant in esthetic regions; ② implant location can be identified; ③ multiple consecutive implants with narrow inter-plant distance.
Modified cross-shaped incision	The horizontal and vertical incisions are located on the side with wider keratinized gingiva around the implant. T-shaped or L-shaped incisions are formed in extreme situations.	Unequal width of keratinized gingiva on the lingual/buccal or proximal/distal sides of the implant.
S-shaped Incision or C-shaped incision	The horizontal incision is along the palatal side of the ridge, and S-shaped or C-shaped incisions are located on the labial gingiva between implants.	① Multiple adjacent implants; ② implant location cannot be determined precisely; ③ enhancement of interproximal soft tissue height.
Split-finger incision	The “finger” flaps are divided and reflected to the respective mesial and distal sides of the implant, each is at least 2.0 to 2.5 mm wide.	① Papilla reconstruction; ② 2 or more adjacent implants.

### 1.4. Papilla preservation flap designs in alveolar surgery

**Figure 3.**
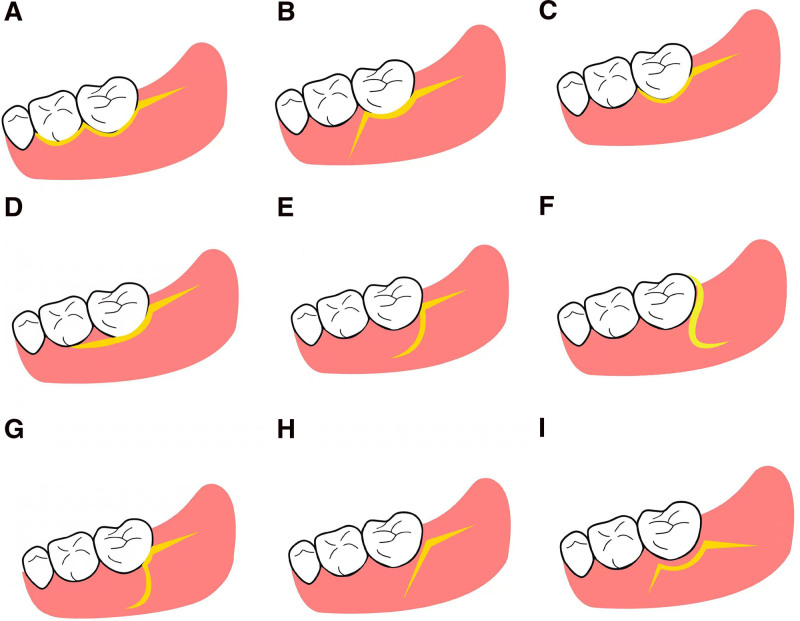
Illustration of the flap designs in impacted mandibular third molar extraction (buccal view). (A) standard envelope flap; (B) standard triangle flap; (C–I) modification flaps.

The flap design is 1 of the main steps in the removal of impacted mandibular third molars (IMTM). The ideal flap designs should provide adequate surgical access, facilitate the suturing, promote the healing, and minimize postoperative reaction to achieve a balance between trauma minimization and convenience maximization. At present, the envelope flap (Fig. 3A) is 1 of the most common flap techniques employed in clinic.^[49]^ In the standard envelope flap, a buccal sulcular incision surrounds the first and second molars to the distal edge of the second molar, continued with a horizontal incision extending distally 1 to 2 cm in the mandibular ramus. The envelope flap is considered the gold standard of flap designs in IMTM, with advantages including ease of suturing, minimal scarring, and mild postoperative swelling and pain.^[50–52]^ However, this procedure impairs the integrity of interproximal papilla between the first and second molars. The standard triangular flap (Fig. 3B), is also 1 of the 2 most favored flap designs,^[53]^ consisting of a horizontal incision and a relieving incision starting from the mesibuccal edge of the second molar. Though damage to the papilla is minimized, its relieving incision extends above the mucogingival junction, thus a heavier postoperative reaction can be expected. Moreover, the relieving incision is difficult to suture, easily inducing wound dehiscence and exposure of the extraction cavity to the oral environment in the early stage, increasing the incidence of alveolitis.

Incisions derived from the above 2 conventional incision designs strive to reach a balance between minimizing postoperative reactions and maximizing periodontal preservation. Several incision techniques with papilla preservation are summarized into 3 types, including modified envelope flaps, modified triangular flaps and other modified flaps.

The modified envelope flaps are represented by the Kruger envelope flap (Fig. 3C),^[54]^ a minimally invasive envelope flap,^[55]^ and the papilla preservation envelope flap (Fig. 3D).^[56]^ These flap designs can effectively solve the problem of papilla damage triggered by the traditional envelope flap. The Kruger envelope flap and the minimally invasive envelope flap independently terminate the intrasulcular incision at the mesiobuccal line angle or the midbuccal surface of the second molar, without the involvement of the first molar. When they are used for IMTM, there is a risk of poor exposure. In the papilla preservation envelope flap approach, the sulcus incision of the second molar is medially continued with a direct horizontal cut through the base of the papilla between the first and second molars.

There have been many reports of modified triangular flaps, such as the Szmyd flap (Fig. 3E),^[57]^ comma flap (Fig. 3F),^[58]^ and bayonet flap (Fig. 3G).^[59]^ The sulcus incision of the Szmyd flap is terminated in the distobucal edge of the second molar, and a relieving incision is subsequently made. When bone removal is enlarged medially, it inevitably leads to no solid bone wall below most incision margins. The comma flap and bayonet flap are similar to the Szmyd flap, with a relieving incision curving forward or backward to the mandibular vestibule, resembling a comma or bayonet. Although the adjacent papilla is protected, more severe postoperative pain and swelling are present due to the release incision exceeding the mucogingival joint. To minimize the impact of flaps on the periodontal health of adjacent second molar, Suarez-Cunqueiro et al^[60]^ and Kirtiloğlu et al^[61]^ advocated placing the incision on the attached gingiva, 1 to 2 mm from the free gingival margin (Fig. 3H and I). The paramarginal modifications have been demonstrated to have superiority in promoting periodontal healing of the adjacent molar.^[62]^ Due to the preservation of the periodontal ligament and attached gingiva around the second molar in paragingival incisions, potential periodontal impairment can be avoided. However, the width of the attached gingiva on the buccal side of mandibular molars is narrow, especially in patients with alveolar bone resorption and gingival recession. This may account for the trend in age-related decreases in keratinization. Therefore, the width of the attached gingiva may not meet the requirements for subgingival incisions. Kirtiloğlu et al^[61]^ and Passarelli et al^[63]^ conducted periodontal probing 1 or 2 weeks post-operatively to assess the short-term periodontal health of the second molar. Although the appearance of the gingiva is restored 1 to 2 weeks after surgery, the junction epithelium formed by connective tissue is fragile. During this time, periodontal probing may damage the reattachment of periodontal fibers, generating large experimental error.

Other modified flaps based on nontraditional gingival flaps, are not typically used in clinical practice. For example, the lingual based triangular flap^[51]^ and lingual based 4 cornered flap,^[64]^ are equally limited by extensive flap reflection, risk of lingual nerve and periodontal damage. The single linear flap,^[65]^ on the other hand, is plagued by a poor surgical field of view and inadequate exposure of IMTM.

Most research investigating the incisions of IMTM removal emphasize their influence on postoperative reaction and operational efficiency. However, less attention has been given to periodontal healing in relation to flap designs, especially preservation benefits of papillary height between the first and second molars adjacent to IMTM.

### 1.5. Papilla preservation flap designs in orthodontic surgery

Periodontally accelerated osteogenic orthodontics (PAOO) refers to a clinical approach through which selective alveolar corticotomy is carried out, in combination with particulate bone grafting for accelerated tooth movement in orthodontic therapy. The PAOO was first proposed by Wilcko et al^[66]^ in 2001, requiring elevation of both labial/buccal and palatal/lingual full thickness flaps using sulcus incision, with extensive decortications of the labial/buccal and palatal/lingual alveolar bone. While this procedure increases orthodontic efficiency, the extensive flap elevations are quite invasive, thereby minimizing adoption of the technique. Viwattanatipa et al^[67]^ introduced a single flap corticotomy (SFC) approach to accelerate tooth movement, observing favorable results. Addanki et al^[68]^ demonstrated in 2017 that both SFC and bilateral flap corticotomy share a similar effect on acceleration of orthodontic tooth movement. According to the above researchers, SFC can reduce surgical trauma when generating great acceleration effect, increasing its routine use in clinical practice.

The philosophy of minimally invasive techniques has led to the constant optimization of the PAOO procedure, as researchers strive to simplify surgical procedures and reducing patient discomfort while ensuring efficacy. Dibart et al^[69]^ proposed the Piezocision technique in 2009. Instead of flap reflection, a longitudinal opening is performed directly to the alveolar bone surface in the buccal attachment gingiva, below the interproximal papillae. Subsequently, a piezoelectric knife is inserted into the longitudinal openings to create the bone injury. In patients with anatomical inadequacies, a subperiosteal tunneling procedure is performed with blunt separation of the longitudinal incision, to accommodate the bone graft or soft tissue graft. However, if no flap is reflected, the space for bone grafting becomes insufficient and the grafting position is difficult to identify.

The computer-aided 3-dimensional surgical guide accurately locates the incision position, length, and depth to develop personalized plans pre-operatively. Ultrasonic osteotome assistance further improves the accuracy and efficiency of the non-flap PAOO procedure.^[70,71]^ Although there is sufficient evidence to suggest that Piezocision technique can accelerate tooth movement, the idea that non-flap techniques can also generate rapid tooth movement has been questioned by Binderman et al.^[72]^ Their study in 2001 found that only reflecting the flap (without decortication) can promote the remodeling of alveolar bone and soft tissue. Further, in 2010, they proposed that fiber incision during flap reflection was the main reason driver of alveolar bone resorption and acceleration of orthodontic tooth movement.^[73]^

Gingival recession is a frequent observation during orthodontic treatment.^[74]^ The traditional PAOO procedure applies intrasulcus incision to raise full thickness flap to expose the entire alveolar bone surface, worsening the atrophy of the gingival papilla post-operatively. Murphy et al^[75]^ recommended that the interproximal tissue should not be detached from the underlying alveolar bone, especially in esthetically sensitive areas, suggesting that incision techniques with retention of the gingival collar could provide collateral blood supply to the papilla. Wang et al^[76]^ designed a modified subgingival sling suture technique for PAOO. In this technique, a scalloped incision was placed 3mm below the free gingival margin, combined with a novel subgingival method for sling suture to close the wound. The suture technique ensures the bulk of the suture is hidden subgingivally, with limited segments exposed to the oral environment, minimizing dental plaque contamination to the bone augmentation and preventing gingival recession. However, the experimental results showed that there was no bone augmentation in the coronal portion of the root after surgery, which is also a common limitation of papilla preservation flap designs. Thus this technique is not suitable for patients with bone defects in only coronal level of the root.

Following the traditional PAOO approach, though an increase in the alveolar bone width at the midroot and apex level can be created, insufficient regeneration at the coronal level was still observed. The surgical procedures and displacement of the graft material may be responsible for this phenomenon. Alveolar bone loss, including bone fenestration or dehiscence, occurs commonly in the midroot and coronal level of anterior mandibular teeth.^[77]^ There is a clear necessity to achieve bone augmentation in the coronal portion. Ma et al^[78]^ proposed the dumpling technique in 2016. The technique employs a horizontal incision at the mucogingival line to dissect split-thickness flap apically to the mental region. The periosteum is incised at the mental region and elevated coronally from the alveolar bone to facilitate the selective alveolar decortications and subperiosteal placement of bone graft material. Subsequently, the periosteum covering the bone graft is attached to the labial alveolar surface with sutures in a dumpling-like fashion. The initial incision at the mucogingival junction provides a better seal for the bone graft around the alveolar crest to protect the grafting materials from displacement and leakage. Moreover, this incision minimizes the suture tension in the coronal region, which benefits the regeneration of horizontal bone thickness at the coronal third of the dental root.

## 2. Conclusion

Currently, papilla preservation has become one of the main periodontal considerations for oral surgery. The maintenance of periodontal contour not only meets the patient’s aesthetic needs, but also supports favorable surgical outcomes while eliminating a series of complications following papilla deficiency. To maintain the periodontal integrity, the incision position has evolved over the years: moved apically from the coronal point to the base of the papilla, as far as the oral mucosa beyond the mucogingival line. The microsurgery technique and dental digitalization have provided narrower margins of surgical access and better facilitated periodontal healing, which may become the trend for papilla preservation technique. The differences in clinical benefits between different papilla preservation flap designs are not fully understood, so more sufficient evidence is needed in the future to clarify their indications and efficacy differences. Further, to clarify the positive effects of papilla preservation technology on periodontal tissue, researchers are encouraged to analyze the structure of plaque microorganisms, the expression of inflammatory factors and related proteins in periodontium of papilla preservation incisions and traditional flap designs from the perspectives of microbiology and host immune response.

## Author contributions

**Conceptualization:** Yinghua Fu, Zhixin Zhang, Jiangling Su.

**Data curation:** Yinghua Fu, Zhixin Zhang, Xiaoping Tang, Jiangling Su.

**Formal analysis:** Yinghua Fu, Zhixin Zhang, Xiaoping Tang, Jiangling Su.

**Funding acquisition:** Jiangling Su.

**Investigation:** Yinghua Fu, Zhixin Zhang, Jiangling Su.

**Methodology:** Yinghua Fu, Zhixin Zhang, Xiaoping Tang, Jiangling Su.

**Project administration:** Yinghua Fu, Zhixin Zhang, Jiangling Su.

**Resources:** Jiangling Su.

**Software:** Yinghua Fu.

**Supervision:** Jiangling Su.

**Validation:** Yinghua Fu, Zhixin Zhang, Xiaoping Tang, Jiangling Su.

**Visualization:** Yinghua Fu, Zhixin Zhang, Jiangling Su.

**Writing – original draft:** Yinghua Fu, Zhixin Zhang, Jiangling Su.

**Writing – review & editing:** Yinghua Fu, Zhixin Zhang, Jiangling Su.
